# The Way Older Childless Women Value Their Life—A Qualitative Study

**DOI:** 10.3390/bs14050418

**Published:** 2024-05-15

**Authors:** Tom Boker Gonen, Yaacov G. Bachner, Vered Slonim-Nevo

**Affiliations:** 1 M.A Program in Gerontology, School of Public Health, Ben-Gurion University of the Negev, Beersheba 8410501, Israel; bachner@bgu.ac.il; 2 The Charlotte B. and Jack J. Spitzer Department of Social Work, Ben-Gurion University of the Negev, Beersheba 8410501, Israel; slonim@bgu.ac.il

**Keywords:** pro-natalism, childless women, life review, locus of control, qualitative study

## Abstract

Older women without children, like all older adults, evaluate their lives and face a conflict between despair and ego integrity as proposed by Erikson’s theory of development. Their uniqueness lies in their deviation from the societal norm of parenthood prevalent in pro-natalist societies such as Israel. This study aims to explore how older childless women evaluate their lives. Using a qualitative approach, 20 semi-structured, in-depth interviews were conducted with women over 60 years of age who do not have children. Three main themes emerged from the participants’ responses: their experiences as women without children in a pro-natalist society, the significance of freedom in their lives, and their life experiences from conflicting perspectives—an internal and external locus of control. The study’s findings demonstrate that older childless women adeptly utilize different perspectives across various aspects of their lives, contributing to ego integrity, contrary to the prevailing societal notion that in the absence of children, women are damaged and lack identity. It is conceivable that other segments of the older adult population, diverging from mainstream societal norms, may similarly leverage these different perspectives to uphold their ego.

## 1. Introduction

Global transformations have altered the concept of the family unit, allowing for the separation of relationships, sexuality, and parenthood. This shift has contributed to a rise in the number of individuals without children [1]. According to the OECD, there is an observable increase in the percentage of women aged 40 to 44 without children. However, it is important to note that existing data are not consistently available, and in many countries, there are a lack of comprehensive data spanning multiple years. In Israel, as of 2008, 10.8% of women aged 40–44 were reported to be childless [2]. Israel is often characterized as a pro-natalist state where childbirth and parenthood are upheld as central values [1]. The policy of encouraging childbirth was rooted in society and established a widespread perception of motherhood as a social status [3]; consequently, there is criticism towards childless women [1]. These women face social stigma [4] and are labeled as being emotionally blocked, damaged, selfish [5], and having a damaged identity [6]. Nevertheless, Rothrauff and Cooney’s study did not find conclusive evidence supporting the notion that parenting is indispensable for the development of women [7].

Among childless women, there is a unique group of older women. While specific data regarding the number of older childless women in Israel are unavailable, U.S. Census Bureau data indicate that 15% of women over the age of 55 are childless in the United States [8]. The State of Israel incorporates fertility treatments into its state health services, extending these services to women until the age of 54 for treatments involving egg cell donation [9]. Beyond the age of 60, the likelihood of natural conception diminishes due to diminished egg cell quality and a depleted reserve of ovarian follicles [10]. The social distinctiveness of older childless women influences their lives. Their identity encompasses membership in various social groups, impacting their self-perception and societal expectations [11]. For example, their gender places them under unique societal expectations regarding family-raising, child education, and household responsibilities [12]. In addition to belonging to a subset of individuals who are not parents, situated within a pro-natalist society [13], they are also a part of the older adult’s demographic in a society with ageist perceptions [14]. The deviation of these women from the social norm of parenting leads to societal labels [5,6,15]. There is a risk that they may internalize these stigmas, potentially impacting their life evaluations and their ability to attain ego integrity detrimentally.

Due to the unique circumstances of older childless women, they encounter distinct challenges in many aspects in their old age. Socially, according to studies, older adults without children have a limited social network compared to those with children [6,16]. The main areas of assistance that the family should provide are emotional support, instrumental help, and contact with health, welfare, and economic institutions. Aging without children decreases the amount of family assistance to older adults, since the children usually provide this assistance [17]. Hsieh and Zhang, found that in the absence of family support, older adults tend to neglect their health problems, which may be a factor that increases mortality [16]. Emotionally, according to Erikson’s theory of development, older adults experience an internal conflict when observing and evaluating their own life. When a person examines his own life and evaluates it positively, the result of the internal conflict will be ego integrity, even if he had difficulties and failures in the past [18]. There are conflicting findings about the emotional state of older adults without children. On the one hand, the study by Vikstrom and others found that older adults without children do not experience loneliness and depression at a higher level than older adults with children. The reason for this may be the use of coping strategies, adopted throughout life, in dealing with their deviation from the social norm [19]. On the other hand, Hsieh and Zhang found that childless older adults report depression, poor wellbeing, loneliness, anxiety, and multiple functional limitations, more than older adults with children [16]. From the scientific literature, it is impossible to assess whether the absence of children affects their emotional state in old age and how they value their lives. In the context of a pro-natalist societal framework, childless women encounter stigmatization due to their departure from the established social norm of parenthood, subsequently being subjected to derogatory labels such as “damaged”. Within the framework of Erikson’s psychosocial developmental theory, childless women in their later years confront a profound existential conflict between despair and ego integrity as they engage in a retrospective assessment of their life trajectories. This study seeks to scrutinize how older childless women value their life experiences within the confines of a pro-natalist society that actively advocates for and emphasizes the imperative of childbirth. While the scientific literature encompasses many studies on individuals without children [1,4,5,15], there is a scarcity of research explicitly focusing on women in old age. 

## 2. Materials and Methods

This study utilized a qualitative research method. While numerous studies have delved into the experiences of childless women [1,4,5,15], this research specifically focuses on this demographic in old age.

### 2.1. Participants

The study’s participants were selected based on their compliance with predetermined criteria in purposive sampling [20], which included women over the age of 60 who were not mothers and were living in Israel. Academic literature lacks a precise definition for old age. For this study, the age of 60 was established as the threshold for participation since some researchers define this age as older adulthood. Childless women over the age 60, are not organized under an official body or institution. The invitation to the study was distributed to social workers in institutions for older adults, such as welfare departments for older adults, assisted living facilities, nursing companies, and day centers. The referral to older adults’ institutions stemmed from the understanding that although most older adults were unsuitable for the study, older adults without children tend to turn more to welfare services to fulfill their needs when family support is limited [6]. Social workers from these institutions facilitated the connection with women who were suitable for the study. Simultaneously, the invitation to participate in the study was published on the social network Facebook in designated groups for seniors and people without children. A description of the study’s participants is presented in Table 1.

### 2.2. Data Collection

The study’s data were gathered through semi-structured, in-depth interviews [21], employing an interview guide approved by the ethics committee of Ben Gurion University (protocol code 39-2022). Initial contact with the participants occurred through a telephone call, during which the purpose of the study and the intention to conduct face-to-face interviews, subsequently recorded, were explained. Regrettably, four women declined to participate, expressing a preference for telephone interviews, leading to their exclusion from the study. The interviews commenced with a general question, “Tell me about yourself” providing participants the opportunity to articulate their narratives and share salient aspects of their lives from their perspectives. Following this, additional questions were posed regarding various aspects of their lives, including their childhood, adulthood, and the experience of not having children. At the end of each interview, additional questions were asked to gather socio-demographic information, including age, family status, and sexual preference. All interviews were conducted face-to-face, with the participant locations determined by the participant’s preferences, recognizing the significance of creating a secure and conducive environment for sharing personal details. Predominantly, participants opted to meet at their residences, while three preferred interviews in a café, two in a park, and one in her office. All interviews transpired in quiet, secluded, and private settings.

### 2.3. Data Analysis

The interviews were analyzed using the thematic analysis method, which refers to the interview as a reflection of the inner world of the participants. In the first phase, all interviews were subjected to a comprehensive analysis, during which initial observations were made regarding the insights derived from the interviews [22]. For instance, a notable contrast emerged between the perception of life as predetermined by fate and the lived experience of having choices and the capacity to shape one’s own life. Subsequently, each interview was carefully reread and categorized into distinct themes based on its content [23]. Following the categorization of interviews, a tabular representation was constructed, with each column corresponding to one interview, listing all pertinent topics derived from it. This table facilitated the visualization of topics across all interviews and enabled the examination of connections between them, leading to the formulation of three central themes [22]. 

### 2.4. Standards for Evaluating the Quality of the Research 

The reliability of the study was strengthened by using direct quotes and rich detail about the procedure of recruiting the participants, conducting the interviews and their location, and the process of analyzing the findings. While conducting the interviews and their analysis, the researcher compiled a researcher’s diary that allowed familiarization with the researcher’s feelings, distinguishing them from the knowledge that emerged from the interviews. This diary provided the researcher with the means to self-examine the research process. Throughout the analysis process, the researchers returned to the interview recordings to maintain the broad context of the interviews and the reliability of the study’s findings. 

### 2.5. Ethical Issues 

The qualitative study involves ethical considerations arising from the interactions between the researcher and the participants, including the dynamics of their relationship. Firstly, to uphold the confidentiality of the participants, pseudonyms were utilized in the study for all names. 

Among the consenting participants, the majority expressed a preference for the interviews to be conducted in their homes, perceived as a space granting researchers access to their private realms. Home settings, however, introduced complexities concerning privacy boundaries, necessitating meticulous care from interviewers to safeguard the confidentiality of participants. Notably, in one instance of the 14 interviews conducted at the participant’s home, the participant’s husband was present, while in 3 others, nursing assistants were in close proximity. All interviews were conducted in a designated private room to mitigate potential influences on interview content and the participants’ openness. At the beginning of the interview, participants were apprised of their prerogative to terminate the interview at any juncture and to solely respond to queries within their comfort zone. Furthermore, the researcher provided the participants with comprehensive contact details, extending an avenue for recourse in the event of post-interview distress. Participants were further informed of their option to defer the interview until the eventual publication of the study. 

It should be noted that in order to enhance and refine the text, some parts were edited for English language improvements using AI tools.

## 3. Results

Three main themes emerged from the participants’ responses regarding how older childless women appreciate their lives: firstly, their experiences as women who are not mothers in a pro-natalist society; secondly, the meaning of freedom in their lives. Thirdly, the participants described events from their lives from contrasting perspectives: on one hand, they described life as designed by fate, and on the other hand, they believed that they can shape their life as they want.

### 3.1. Experience as Childless Women in a Pro-Natalist Society 

The participants described their lives as childless women in a society that sanctifies childbirth and parenthood. They described the events that led them not to be mothers, their feelings about it at old age, and their perception of parenting in the pro-natalist society, as women who are not mothers.

#### 3.1.1. Explanation Provided for the Reasons Participants Are Not Mothers 

The life events that led the participants not to be mothers were different and stemmed from their life experiences, the environment in which they grew up and the opportunities that came their way. Two key aspects of why they were not mothers were identified. Firstly, many expressed difficulty in finding a suitable partner with whom to co-parent. Secondly, some chose not to become single mothers, which they perceived as a direct continuation of the difficulty of finding a suitable relationship. A relationship symbolizes partnership and sharing the effort required in raising a child. Several explanations were identified for the difficulty of finding a relationship for parenting. Firstly, a general difficulty in finding a relationship was noted, as 14 of the participants were single and were never married.

*“I was looking for a partner, in Kibbutz Ha’ir, I went out to the city for a bit, came back to the farm, uh... I couldn’t find the partner”.*

*(Orit)*

The second difficulty was having a relationship that is not suitable for parenting. Not every relationship is well-established and stable enough for partnership and long-term commitment. Rinat described that she had several relationships that were not suitable for parenthood. In some cases, the man made it clear that he was not interested in parenting, and in some cases, she decided that she was not interested in parenting with the man with whom she was in a relationship.

*“All the relationships that I had, and had, stood in contradiction to... the children [...] either they didn’t want, or I looked at the man I was with and I said that I’m not ready for him to be the father of my children, and I’m not ready for a child who can’t be returned to the store to bind us forever”.*

*(Rinat)*

In contrast to Rinat, Amalia described a relationship dedicated to parenting that did not achieve this goal. Towards the age of 40, Amalia decided to try to be a mother and found a partner who was interested in parenting like her. Despite the desire of the two, Amalia was unable to conceive, and the relationship eventually ended.

*“I said, well, he can only suit me if I conceive, only. And it’s been a whole year, and I didn’t, and I got tested and the doctor told me, ‘You are perfectly fine’. The doctor told me that my partner needed to do a test [...] I told him that if he didn’t have test results when I was back, he shouldn’t be here. And so it was, I knew he wouldn’t, probably he knew he had some kind of an issue”.*

*(Amalia)*

In addition, the participants described their choice not to be single mothers due to the physical and mental strength required in raising a child alone and the belief that a child needs two parents. Their choice not to be single mothers was influenced by the difficulty of creating a relationship in general and parenthood in particular.

*“I thought about being a single mother and I thought that for the child, from my point of view, a child needs two parents. It’s a big job if you take it seriously and... I didn’t think I had enough strength, mentally and physically, to take care of the child myself. So, I said to myself, ‘I will not raise a child alone’”.*

*(Mimi)*

#### 3.1.2. Between Regret and Reconciliation

The participants expressed a range of emotions regarding their non-motherhood status. Kamila described regret; however, Sigal described an acceptance regarding her status.

*“What I regret in life is that I don’t have children”.*

*(Kamila)*

*“I’m neither a mother nor a father ((laughing)), yes... uh... I don’t mind that. I don’t mind, no [...] I don’t have any... uh... I don’t have any Ah... Deliberations or... I don’t have any... qualms of conscience”.*

*(Sigal)*

While some expressed regret and some described a feeling of completeness and acceptance with this aspect of their lives, some women described a change in their feelings that resulted from the ability to examine the past. Yochi described both regret as well as acceptance that she is not a mother. These feelings, which usually oppose each other, testify to the process she went through over the years. She wanted to be a mother, but it did not happen. Today, at 71 years old, she has no regrets about the way her life turned out.

*“Let’s say that up until the age of 30, I actually thought about having a child and it didn’t work out... now I don’t regret it... in retrospect, I don’t regret it”.*

*(Yochi)*

On the other hand, Kira expressed deep sorrow for not having children. Kira’s regret characterized her mature life; according to her, the change occurred because she grew up and could examine her past.

*“I’m quite sorry for the process I went through […] I’m not satisfied with the fact that I wasn’t a mother [...] But everything is in today’s view because today I say sorry, but then I was in a completely different situation”.*

*(Kira)*

#### 3.1.3. Criticism towards Parenting 

The participants described their points of view regarding parenting, including the motives for parenting and children’s behavior towards their parents. Some of the participants believed that parenting stems from the parent’s egoistic motives. Yochi described her point of view as one in which parenthood fulfills the parent’s needs and is not necessarily intended to benefit the child.

*“I think that having a child... I mean, I also think, for example, that parenthood is a very selfish step. He doesn’t bring the... he wants the child for himself, and not for the child”.*

*(Yochi)*

Another criticism refers to the failure of the children to fulfill their responsibilities to their parents. Parents face difficulty when their children become independent, sometimes without gratitude for the parental effort in raising them. From the eyes of the participants, parents in old age feel that their children have forgotten them.

*“The days you are alone happens to everyone, not only to those who are alone and don’t have children... it also happens to those who have children, and the children don’t always come to visit them... and they are miserable”.*

*(Sigal)*

### 3.2. Freedom 

The participants emphasized their desire for freedom and the resources they invested to achieve it, even when it meant facing social and personal challenges. Freedom allows one to act according to personal needs and desires, without the need to be flexible to the will of others. Commitment, in its various forms, may affect freedom due to the needs of another person. Fourteen of the participants were not married, an aspect that allowed them, from their perspective, to maintain their independence.

*“Being free for me... it’s not just that I’ve never married [...] my freedom is a very, very important value in my life.”*

*(Rinat)*

### 3.3. Different Perspectives on Events in Life

The participants recounted events from their lives through two conflicting perceptions. Firstly, they viewed life as a product of their choices, decisions, and actions, wherein they had the ability to shape their reality, strive towards their goals, and determine the course of their lives. Secondly, some perceived life as predetermined by fate or a higher power, without their ability to exert control. Interestingly, most participants held both perceptions simultaneously, despite their contrast. For instance, Rinat highlighted her experience of not having children. On one hand, she described her proactive choice to undergo fertility treatments in an attempt to conceive and become a mother.

*“And I said OK, so now I’m going to have a child alone like they do [...] I said well I’m going to do it, and I went to do it”.*

*(Rinat)*

On the other hand, she attributed the failure to conceive to “God”, conceptualizing it as a decree by a higher power.

*“The universe helps me decide, I leave it to God, I’m not a religious person but I have a lot of faith. I leave it to God...if God wants it will be successful before the age of 45”.*

*(Rinat)*

Galia also presented conflicting points of view on events in her life. Most of the time, she presented her life as the product of actively choosing how she would live.

*“The labor [...] I said to myself ‘I’m not going through this, I don’t want to go through this.’ […] And I think that then, at the age of 20 or so, I already felt that I was probably not into it. And... over the years, I wasn’t into getting married either”.*

*(Galia)*

At the same time, she described a belief in “*fate*” and “*the universe*” as having the power to shape her life and to open and close opportunities for her.

*“I believe in fate, in the universe [...] late in my life, someone from above is looking down on me and constantly organizing and arranging things for me”.*

*(Galia)*

First, Galia described her choices of not becoming a mother and not getting married as choices that she made and shaped her life. At the same time, she described her belief “*in fate, in the universe*” a belief that another power shapes her life, creates possibilities for her, and directs the path of her life.

## 4. Discussion

This study aimed to understand the subjective evaluations of life among older childless women. In the context of Israel, a pro-natalist nation, women who remain childless are often perceived as deviating from the prevailing social norm [1]. This deviation is often accompanied by social labeling, which can significantly influence self-perception. The impact of such labeling may be either positive, reinforcing one’s sense of self, or negative, leading to introspection and questioning of identity [3]. This study specifically focused on older women without children, investigating how they evaluate their lives in light of societal norms and expectations. 

The study’s findings reveal three main aspects of the way old childless women evaluate their lives. Two primary factors contribute to their childlessness. Firstly, many expressed difficulties in finding a suitable partner with whom to co-parent. Among the 20 participants, 14 remained single in their later years. Secondly, some chose not to become single mothers, which they perceived as a direct continuation of the difficulty of finding a suitable relationship. In view of those decisions and other aspects of their lives, older childless women highlight the significance and centrality of freedom in their life trajectory. Lastly, the study’s findings reveal the different perspectives used by older childless women when evaluating their life and the decisions they made. 

According to Erikson’s developmental theory, a crucial aspect of the aging process involves individuals evaluating their past lives and confronting a conflict with outcomes of either despair or ego integrity [18]. Butler posited that this life-evaluation process is a natural and healthy occurrence that happens universally in old age. This cognitive and conscious retrospective analysis involves revisiting unresolved conflicts from the past and reassessing them through a contemporary lens [24]. In Erikson’s framework, a positive resolution to this evaluative process results in ego integrity, whereas feelings of frustration and regret towards past experiences may lead to a sense of despair [18]. Older childless women employ diverse perspectives when evaluating various facets of their lives. On one hand, they manifest an internal locus of control, signifying a belief in their ability to shape the trajectory of their lives [25]. Accordingly, they articulated instances where they actively directed the course of their lives. Conversely, an external locus of control is evident, reflecting a belief that life is influenced by fate or a higher power as God [25]. This perspective is associated with experiences wherein certain aspects of their lives were perceived as beyond their control. Notably, most of the participants used both perspectives, despite the inherent dissonance between them, navigating the nuanced interplay between them. Their tendency to attribute successes and achievements to an internal locus of control, thereby associating positive facets of their lives with a sense of pride and meaning, is noteworthy. The utilization of an internal locus of control is recognized for its role in fortifying one’s sense of self [26]. Conversely, when recounting failures or expressing regrets, older childless women tended to attribute those to external factors such as fate or a higher power, framing them as circumstances beyond personal control. This deployment of an external locus of control serves as a mechanism for distancing themselves from negative aspects of their lives. The findings underscore the salience that older childless women attribute to the concept of freedom in their lives. These women emphasized the significance of freedom in shaping their life trajectories and as a foundational element influencing their choices. The prominence of freedom in the lives of childless women aligns with findings from prior research [27,28]. The ability to live a life characterized by freedom is perceived as a valuable resource. It serves as a coping mechanism when addressing life’s challenges, such as the experience of being single or lamenting the absence of motherhood among some women. It is conceivable that the interpretation of older women without children regarding the concept of freedom within their lives may stem from a concerted effort to achieve ego integrity during their “life review” phase. The perceived benefits and opportunities engendered by this freedom may serve as a protective mechanism for their sense of self, thereby reducing the likelihood of encountering despair in old age.

The study’s findings reveal the deliberate choice of older childless women to refrain from becoming single mothers. Consistent with the findings of the study by Chauhan and colleagues, the present results highlight that the lack of suitable relationships serves as a key factor influencing women’s decisions to refrain from becoming mothers [29]. These women highlighting that their status as childless women originated from a conscious decision rooted in an internal locus of control. This decision-making process entailed a thorough evaluation of the advantages and disadvantages associated with single parenthood in comparison to a life without children. Historically, societal norms have reflected a prevailing notion that a single parent could not adequately fulfill all the needs of a child, contributing to the non-acceptance of single parenthood. This facet also emerged prominently within the findings of the study, wherein the entrenched social norm emphasizing the necessity of a couple in parenting influenced the participants’ perceptions of competence. While many women affirmed their decision not to pursue single motherhood, some expressed regret regarding this choice. The ability to make such decisions is imbued with elements of power and control over one’s life, accompanied by a responsibility for the ensuing outcomes. Consequently, the women articulated a nuanced interplay of autonomy and control [30] alongside feelings of regret and, at times, perceived failure, which may give rise to a sense of despair when reflecting on their past lives. It is possible that some of the criticism expressed by the participants towards parenting may serve as a means of protecting themselves against feelings of regret for not being mothers. 

The study’s findings revealed that older childless women employ various perspectives to describe events in their lives, thereby enhancing ego integrity. Professionals involved in facilitating “life review” processes for older adults should be aware of these diverse perspectives, including the interplay between internal and external locus of control, and their significance in fostering ego integrity. Furthermore, it is conceivable that other segments of the older adult population, diverging from mainstream societal norms, may similarly leverage these different perspectives to maintain their integrity in the context of aging. Older childless women may encounter societal reactions in a pro-natalist society due to their unique status, deviating from the prevailing societal norm of parenthood. This divergence can give rise to the emergence of social stigmas and attendant challenges [1]. The risk associated with stigma lies in its potential internalization by the stigmatized individual [31]. Consequently, the risks associated with societal stigma toward older childless women encompass their internalization as damaged individuals [5], selfish [15], or considered underdeveloped [6]. As part of the normative “life review” process in old age, older childless women engage in a comprehensive evaluation of their lives. They employ an internal and external locus of control, strategically leveraging these perspectives to bolster positive aspects and navigate away from failures and disappointments. The strategic utilization of both internal and external locus of control serves as a tool, allowing these women to evaluate their lives in a way conducive to preserving ego integrity and avoiding experiences of despair in their later years.

### Limitations

This study has several limitations. The study relied on a small qualitative sample comprising semi-structured in-depth interviews, with 20 women. It does not differentiate between women who were not mothers by choice due to ideological reasons and those who were not mothers due to life circumstances, such as difficulties in finding a partner. There is a difficulty in making a distinction regarding how much women choose not to be mothers [32]; participants testified that throughout life, their perception changed, and some of them did not know how to testify clearly as to why they are not mothers. Moreover, the findings of the study hold relevance within the context of a pro-natalist society characterized by both covert and overt pressures towards parenthood. However, it is imperative to acknowledge the dynamic nature of societal attitudes and norms surrounding parenthood, especially considering the ongoing global shifts in social perceptions and behaviors related to childbearing. Thus, while the research outcomes offer valuable insights, their applicability to contemporary cohorts of young childless women may warrant closer examination and potential recalibration.

## 5. Conclusions

Older childless women in a pro-natalist society attribute meaning to their lives and portray them positively, even when discussing failures, regrets, and a sense of missed opportunities from their past. They achieve this by harmoniously employing both internal and external locus of control in their narratives. This nuanced utilization of cognitive mechanisms allows them to navigate and reconcile potential conflicts, contributing to a more positive overall evaluation of their lives within the context of societal expectations.

## Figures and Tables

**Table 1:** Description of the Study’s participants.

Name (Pseudonym)	Age	Place of Living	Level of Religiosity	Sexual Preference	Family Status	Health Condition
Galia	78	City	Secular	Heterosexual	Single	In good health
Shlomit	63	City	Secular	Heterosexual	Single	In good health
Mimi	72	City	Secular	Heterosexual	Married	Recovering from cancer
Yochi	71	City	Secular	Heterosexual	Single	In good health
Flora	95	City	Secular	Heterosexual	Widow	Have a nursing assistant
Tirzza	76	Kibbutz	Secular	Heterosexual	Single	In good health
Hava	80	City	Secular	Heterosexual	Single	In good health
Sigal	72	City	Secular	Heterosexual	Single	In good health
Ana	61	City	Secular	Heterosexual	Single	In good health
Kira	65	City	Secular	Heterosexual	Single	In good health
Ilana	67	City	Secular	Heterosexual	Widow	In good health
Rinat	65	City	Secular	Heterosexual	Single	In good health
Orit	64	City	Secular	Heterosexual	Single	In good health
Oxana	69	City	Secular	Heterosexual	Widow	Have a nursing assistant
Shoshana	72	City	Secular	Heterosexual	Divorced	In good health
Svatlana	76	City	Secular	Heterosexual	Married	In good health
Amalia	66	City	Secular	Heterosexual	Single	In good health
Rivka	85	City	Traditional	Heterosexual	Single	In good health
Mari	76	Village	Secular	Heterosexual	Single	In good health
Kamila	78	City	Secular	Heterosexual	Single	Have a nursing assistant

## Data Availability

The data presented in this study are available on request from the corresponding author due to privacy concerns and confidentiality of the interviewees.
